# National Trends in the Ambulatory Treatment of Hypertension in the United States, 1997-2012

**DOI:** 10.1371/journal.pone.0119292

**Published:** 2015-03-04

**Authors:** Meijia Zhou, Matthew Daubresse, Randall S. Stafford, G. Caleb Alexander

**Affiliations:** 1 Department of Epidemiology, Bloomberg School of Public Health, Baltimore, Maryland, United States of America; 2 Center for Drug Safety and Effectiveness, Johns Hopkins Bloomberg School of Public Health, Baltimore, Maryland, United States of America; 3 Program on Prevention Outcomes and Practices, Stanford Prevention Research Center, Stanford University School of Medicine, Stanford, California, United States of America; 4 Division of General Internal Medicine, Johns Hopkins Medicine, Baltimore, Maryland, United States of America; INRCA, ITALY

## Abstract

**Importance:**

Hypertension is common and costly. Over the past decade, new antihypertensive therapies have been developed, several have lost patent protection and additional evidence regarding the safety and effectiveness of these agents has accrued.

**Objective:**

To examine trends in the use of antihypertensive therapies in the United States between 1997 and 2012.

**Design, Setting and Participants:**

We used nationally representative audit data from the IMS Health National Disease and Therapeutic Index to examine the ambulatory pharmacologic treatment of hypertension.

**Outcome Measures:**

Our primary unit of analysis was a visit where hypertension was a reported diagnosis and treated with a pharmacotherapy (treatment visit). We restricted analyses to the use of six therapeutic classes of antihypertensive medications among individuals 18 years or older.

**Results:**

Annual hypertension treatment visits increased from 56.9 million treatment visits (95% confidence intervals [CI], 53.9–59.8) in 1997 to 83.3 million visits (CI 79.2–87.3) in 2008, then declined steadily to 70.9 million visits (CI 66.7–75.0) by 2012. Angiotensin receptor blocker utilization increased substantially from 3% of treatment visits in 1997 to 18% by 2012, whereas calcium channel blocker use decreased from 27% to 18% of visits. Rates of diuretic and beta-blocker use remained stable and represented 24%–30% and 14–16% of visits, respectively. Use of direct renin inhibitor accounted for fewer than 2% of annual visits. The proportion of visits treated using fixed-dose combination therapies increased from 28% to 37% of visits.

**Conclusions:**

Several important changes have occurred in the landscape of antihypertensive treatment in the United States during the past decade. Despite their novel mechanism of action, the adoption rate of direct renin inhibitors remains low.

## Introduction

Hypertension is an increasingly prevalent and costly risk factor for cardiovascular disease, the leading cause of death in the United States [1]. One in every three U.S. adults has hypertension, which corresponds to approximately 78 million people nationwide, yet many hypertensive patients remain undiagnosed [2]. In 2009, the estimated direct and indirect cost of hypertension exceeded $50 billion dollars. By 2030, it is estimated that the prevalence of hypertension will reach 41% [2] and total annual costs will reach $343 billion dollars [3], fueled in part by a growing population with obesity in the United States.

Despite widespread under-treatment, there is some evidence that treatment rates for hypertension have increased during the past decade. For example, in analyses of the National Health and Nutrition Survey (NHANES), Gu and colleagues found that use of hypertension medication among diagnosed hypertension patients increased from 64% (2001–2002) to 77% (2009–2010) [4,5]. By contrast, an investigation using the National Ambulatory Medical Care Survey (NAMCS) found the proportion of hypertension visits treated with an antihypertensive remained stable from 1993 (74%) to 2004 (70%)[6].

Despite insights from these studies, several questions remain, including how the publication of the Seventh Report of the Joint National Committee on Prevention, Detection, Evaluation, and Treatment of High Blood Pressure (JNC 7) and the availability of generic medications have affected treatment patterns. Furthermore, in 2007, the FDA approved Aliskiren, a first-in-class direct renin inhibitor representing the first novel therapeutic anti-hypertensive class approved by the agency in thirteen years. Although results from three clinical trials and several observational studies supported Aliskiren’s effectiveness in reducing blood pressure [7,8,9,10,11,12], evidence also suggests Aliskiren is associated with higher rates of adverse events compared with placebo in diabetic patients concomitantly treated with ARBs or ACE inhibitors [13].

We used nationally representative audit data from office-based physicians to examine trends in the pharmacologic treatment of hypertension from 1997 to 2012. We examined utilization trends within six therapeutic classes: angiotensin receptor blockers (ARBs), calcium channel blockers, angiotensin converting enzyme (ACE) inhibitors, beta-blockers, diuretics and direct renin inhibitor, as well as fixed-dose combination therapies within each class. In addition to examining changes in the use of specific classes of therapies, we also explored whether there have been changes in time in the intensity of treatment and the average age (referred to throughout as vintage) of therapies used.

## Materials and Methods

### Data

We used data from the IMS Health National Disease and Therapeutic Index (NDTI) to examine trends in antihypertensive utilization. The NDTI is a monthly audit of office-based physicians that provides nationally representative data regarding patterns of disease treatment. IMS Health uses the American Medical Association and the American Osteopathic Association master list to select a random sample of more than 4,000 physicians stratified by geographic region and specialty. Participating physicians report information regarding diagnoses and treatments for patients seen during a consecutive two-day period each calendar quarter. The majority of encounters included in the NDTI are office-based visits, however a small proportion are physician visits to patients in long-term care institutions (3%-5%) and hospitals (10%). We excluded these visits in order to focus our analysis on ambulatory practice. For each patient, the participating physician reports all applicable diagnosis and the medications ordered or mentioned to treat each condition. A six-digit taxonomic code capturing diagnostic information similar to International Classification of Diseases 9th Revision (ICD-9) was linked to each record of a drug therapy. Although the sample construction and study design differ, the NDTI provides an audit that is similar to the National Ambulatory Medical Care Survey (NAMCS), which is conducted by the National Center for Health Statistics. Analyses comparing the NDTI with the NAMCS suggest they are comparable in scope [14,15,16].

### Analysis

We used descriptive statistics to examine utilization patterns of each antihypertensive drug class. Our primary unit of analysis included visits where physicians diagnosed hypertension and prescribed one or more medications to treat hypertension (treatment visit). We aggregated quarterly data from 1997 to 2012 to present annual trends. Since many therapies are dispensed as fixed-dose combination products, except where noted we treated a single fixed-dose combination product as counting towards each of its constituent parts; thus, the fixed-dose combination product lisinopril/hydrochlorothiazide counted once towards ACE inhibitors and once towards diuretics. We obtained the mean number of therapies per patient per year by dividing the total number of hypertension treatment visits with each individual therapy by the total number of treatment visits with any prescription. We calculated 95% confidence intervals for our estimates using tables of relative standard errors that account for the stratified sampling design of each annual audit. We also examined the vintage of drugs used during a given year, which we defined as the weighted average of the ages of medications of the six therapeutic classes under study (time since the launch date of the active ingredient of the medication recorded in the NDTI) associated with hypertension treatment visits using December 31^st^ as an annual reference point. We focused our analyses on molecules rather than branded versus generic products, as patients may switch to a generic products when filing their prescriptions.

## Results

### National trends in hypertension pharmacological treatment

Treatment visits for hypertension increased from 56.9 million [M] visits in 1997 to 83.3M in 2008, but then declined steadily to 70.9M by 2012. During the period examined, hypertension treatment visits consistently accounted for 10–11% of all office-based treatment visits (Table 1). The average vintage of medications continuously increased from 17.5 years (1997) to 29.5 years (2012).

**Table 1:** Annual number of hypertension visits in the United States, 1997–2012.

Years	1997	1998	1999	2000	2001	2002	2003	2004	2005	2006	2007	2008	2009	2010	2011	2012
Total treatment visits (Millions)	522	579	583	637	633	664	694	705	739	752	731	734	722	690	666	652
Hypertension treatment visits (Millions)	56.9	61.6	63.1	69.1	65.9	70.4	76.0	79.0	81.0	82.3	81.9	83.3	81.0	75.2	72.6	70.9
Hypertension treatment visit, %	10.3	10.6	10.8	10.9	10.4	10.6	11.0	11.2	11.0	10.9	11.2	11.4	11.2	10.9	10.9	10.9
Average Vintage (Years)	17.5	18.1	18.6	19.3	20.0	20.5	22.1	23.0	24.1	25.1	25.8	26.4	27.0	27.7	28.4	29.5

Source: IMS Health National Disease and Therapeutic Index, 1997–2012.

Table 2 depicts the ten most frequently prescribed individual medications every other year from 1998 to 2012. Amlodipine and lisinopril were the most commonly prescribed medications over the period examined, while no single therapeutic class predominated among these commonly used agents. Fixed-dose combination agents, particularly ACE-inhibitors or ARBs combined with diuretics, were increasingly common during the latter years of observation.

**Table 2:** Most frequently used drugs during hypertension treatment visits, 1998–2012.

	1998	2000	2002	2004	2006	2008	2010	2012
1	Lisinopril	Amlodipine	Amlodipine	Amlodipine	Lisinopril	Lisinopril	Lisinopril	Lisinopril
2	Amlodipine	Lisinopril	Lisinopril	Lisinopril	Metoprolol	Metoprolol	Amlodipine	Amlodipine
3	Atenolol	Atenolol	Atenolol	Metoprolol	HCTZ	Amlodipine	Metoprolol	Metoprolol
4	HCTZ	HCTZ	Metoprolol	HCTZ	Amlodipine	HCTZ	HCTZ	HCTZ
5	HCTZ/Triamterene	HCTZ/ Triamterene	HCTZ	Atenolol	Atenolol	Atenolol	HCTZ/Lisinopril	HCTZ/Lisinopril
6	Verapamil	Metoprolol	Quinapril	Amlodipine/Benazepril	Amlodipine/Benazepril	Valsartan	Atenolol	Losartan
7	Diltiazem	Diltiazem	Amlodipine/ Benazepril	HCTZ/ Triamterene	Valsartan	HCTZ/Lisinopril	Valsartan	Atenolol
8	Enalapril	Quinapril	HCTZ/ Triamterene	Valsartan	HCTZ/ Triamterene	HCTZ/Valsartan	HCTZ/Valsartan	Valsartan
9	Nifedipine	Verapamil	Valsartan	Diltiazem	HCTZ/Valsartan	HCTZ/Triamterene	HCTZ/Triamterene	HCTZ/Triamterene
10	Metoprolol	Furosemide	Diltiazem	Ramipril	Diltiazem	Amlodipine/Benazepril	Olmesartan	HCTZ/Valsartan

HCTZ = hydrochlorothiazide; Amlodipine/Benazepril and other drug combinations with “/” represent fixed dose combination products

Source: IMS Health National Disease and Therapeutic Index, 1998–2012.

**Fig 1:**
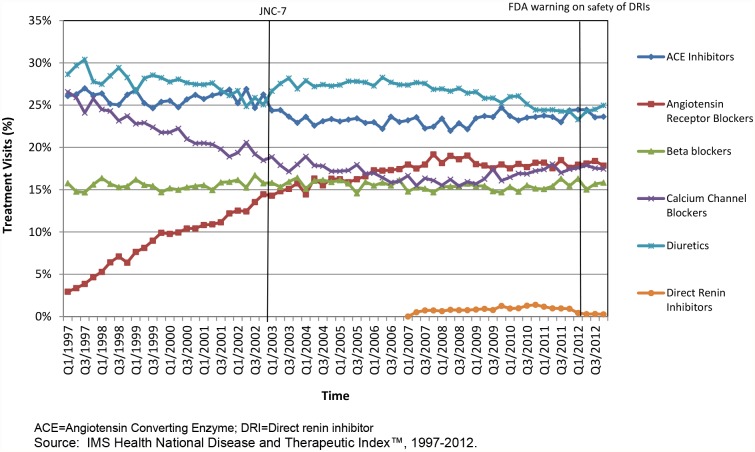
National trends in the use of six therapeutic classes to treat hypertension in the United States, 1997–2012. The utilization of ARBs increased substantially from 3% of visits in 1997 to 18% of visits in 2012. From 1997 to 2008, use of calcium channel blockers declined moderately from 26% to 16%, then increased slightly to 18% of visits by 2012. Trends in diuretic (representing 24–30% of visits) and beta blockers (14–16%) use remained stable.

Fig. 1 characterizes trends in the proportion of hypertension visits treated with each therapeutic class of interest. The utilization of ARBs increased substantially from 3% of visits in 1997 to 18% of visits in 2012. From 1997 to 2008, use of calcium channel blockers declined moderately from 26% to 16%, then increased slightly to 18% of visits by 2012. Trends in diuretic (representing 24–30% of visits) and beta blockers (14–16%) use remained stable. Direct renin inhibitor were rarely used since their market introduction in 2007 and accounted for fewer than 2% of treatment visits during any given year. From 1997 to 2003, the average number of therapies per patient increased from 1.47 (1997) to 1.54 in (2003), and declined steadily to 1.43 therapies per patient by 2012. The fraction of treated patients with only one medication declined from 45% in 1997 to 40% in 2003, then increased to 47% by 2012.

### Hypertension treatment using fixed-dose combinations

**Fig 2:**
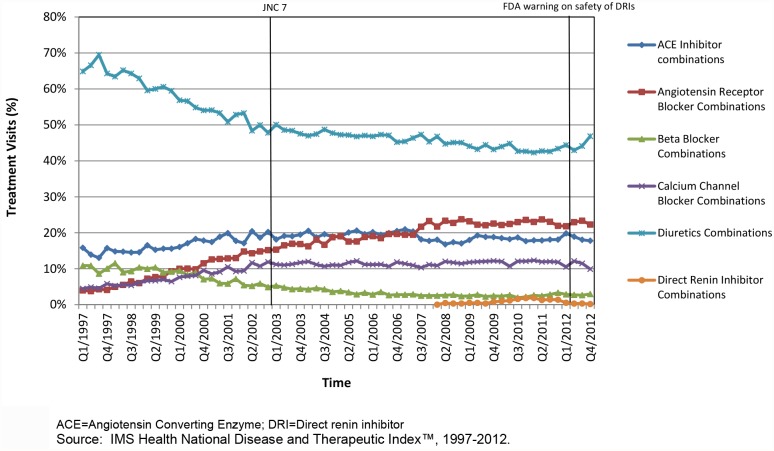
National trends of hypertension treatment visits with fixed-dose combination therapies containing, 1997–2012. Although providers consistently prescribed diuretic combinations more commonly than other fixed-dose combinations, diuretic combination utilization decreased from 65% of fixed-dose combination treatment visits in 1997 to 47% in 2012.

The proportion of hypertension visits treated with fixed-dose combination therapies increased from 28% in 1997 to 41% in 2009 and declined slightly to 37% by 2012. Although providers consistently prescribed diuretic combinations more commonly than other fixed-dose combinations, diuretic combination utilization decreased from 65% of fixed-dose combination treatment visits in 1997 to 47% in 2012 (Fig. 2).

Utilization rates of ARB fixed-dose combinations increased substantially from 4% of fixed-dose combination treatment visits in 1997 to 23% of visits in 2008, then remained constant until 2012. Similarly, the use of fixed-dose combination products containing CCBs increased from 5% (1997) to 11% (2002) of fixed-dose products and then stabilized. During the time period examined, ACE inhibitor combination utilization remained stable ranging from 14% to 20% of treatment visits associated with a fixed-dose combination therapy. Fixed dose combinations containing direct renin inhibitor were seldom used in clinical practice.

Before the debut of the ARB/calcium channel blocker (CCB) combination drug in 2007, the ACE inhibitor/CCB combination was the only combination drug that did not contain diuretics and was being increasingly prescribed by physicians. After 2007, use of ACE/CCB combination drugs declined and ARB/CCB combinations became more commonly prescribed.

## Discussion

Using a nationally representative audit of ambulatory practice from 1997 through 2012, we found increases in use of ARBs and fixed-dose combination products in the United States. We also identified reductions in the utilization of calcium channel blockers, relatively unchanged use of diuretics and beta-blockers, and persistently low rates of direct renin inhibitor utilization in ambulatory practice. Increasing patent expiry and fewer new products have led to an increase in the average vintage of therapies.

Antihypertensive medications will play an increasingly important role in clinical practice during the coming decades, given that one-third of U.S. adults are hypertensive [2]. Our findings suggest that hypertension was reported as a diagnosis in more than a tenth of all physician office-visits. Although the rate of blood pressure control had increased substantially during the 1990’s [17] and 2000’s [4], the prevalence of hypertension is projected to increase [3]. Guidelines from JNC-7 recommend diuretics for most patients as initial therapy for hypertension, however use of ARBs, calcium channel blockers, beta blockers, and ACE inhibitors may also be appropriate based on compelling clinical indications [18]. Our analysis of individual therapies suggests the diversified use of various classes of agents. Multiple factors may have contributed to the observed changes in prescribing patterns over time, including changes in disease prevalence, blood pressure control, publication of clinical guidelines, pharmaceutical marketing and promotion, availability of generics as well as the introduction of new therapies and scientific evidence. Alpha blockers, which lower blood pressure by selectively blocking post-synaptic alpha-adrenoreceptors to prevent vessel restriction, are indicated as add-on therapy to most other antihypertensive therapies [19] and because of their relatively infrequent use [5] for the treatment of hypertension we excluded these from our analyses. We also excluded other antihypertensive medications such as central agents and direct vasodilators, which accounted for only a small proportion of all treatment visits.

The Eighth Joint National Committee (JNC 8) recently published its 2014 Evidence-Based Guideline for the Management of High Blood Pressure in Adults. The report recommends the selection of initial therapy among four classes of medications, including ACE inhibitors, ARBs, calcium channel blockers and diuretics for the general non-black population and updates previous recommendations for specific subgroups [20]. The shift to a more lenient systolic blood pressure goal may delay the initiation of hypertension management using pharmacotherapies, but the impact of this new guideline on practice patterns will ultimately depend on a variety of factors that affect practitioner and patient knowledge, attitudes and behavior [21].

Our finding of low rates of adoption of direct renin inhibitors is consistent with studies of the use of these agents outside of the United States [22]. Despite representing a novel mechanism of action, there are many factors that influence the clinical adoption of a new therapy after market debut, including the number of therapies currently available on the market, as well as their safety, tolerability and level of marketing and promotion [23]. In the case of Aliskiren, the availability of numerous other antihypertensive classes, high costs and safety concerns among select subpopulations such as diabetics [13]; have likely contributed to its low uptake.

We found increasing use of fixed-dose combinations and although the utilization of diuretic combinations decreased, they remain the most commonly prescribed combination antihypertensive therapy. Fixed-dose combination products offer greater convenience compared to single-molecule therapies and may be associated with greater adherence [24] and blood pressure control [25]. On the other hand, the out-of-pocket cost of branded fixed-dose combination products often exceeds the costs of their constituent parts, and thus the clinical benefits of brand-named fixed-dose combination antihypertensive therapy have to be balanced with their greater economic burden for patients [26]. For certain drug makers, the creation of combination therapies has served as a successful strategy to extend their patent protected market[27] and most of these fixed-dose combinations have become generically available, except for some ARB/CCB combinations and two or three drug combinations containing direct renin inhibitors. The lower cost of these generic fixed-dose combination products may also contribute to increases in their use of over time.

Our findings of a decline in fixed-dose ACE/CCB combinations use after 2007 and a decline in amlodipine/benazepril combination use was consistent with the time period when the most commonly prescribed branded ACE/CCB combination, Lotrel, went generic in 2008. Combined use of ACE inhibitors and CCBs as free combinations may still be common after 2008, given both amlodipine and lisinopril remained one of the most frequently prescribed agents.

Although there were modest increases in intensification of therapy over time, the average number of products per patient was similar in 2012 as it was in 1997. This is noteworthy given the societal burden of undertreated hypertension, as well as the fact that the use of two or more hypertensive agents improves patient response[25], and the presence of JNC-7 guidelines that recommend the use of two agents from multiple classes for patients with stage two or more severe hypertension [18].

Our findings of increased antihypertensive use are similar to a population-based analysis of the National Health and Nutrition Examination Survey that examined treatment patterns from 2001–2010[4], although in contrast to this prior study, we found decreasing rather than stable rates of calcium channel blockers use and stable rather than increasing use of ACE inhibitors over a longer period of time. These differences are likely due to variation in the study design of these contrasting surveys; the NHANES is a population-based survey of patients reflecting use of medication at the individual-level, whereas the NDTI represents a visit-based sample and reflects clinician-reported uses of specific therapies [4].

Our study has several important limitations. First, the NDTI provides aggregated population-level estimates, thus we were unable to examine individual-level decision-making such as treatment initiation, discontinuation, or switching between antihypertensive agents. Second, the NDTI provides limited clinical information such as patients’ blood pressure, JNC-7 class, or other measures of clinical severity required to judge the clinical appropriateness of prescribed therapies. Finally, our analysis focuses on provider behavior in ambulatory practice, which does not reflect prescription dispensing or adherence. While we observed a decline in treated hypertension visits in recent years, this corresponded to an even greater proportional decline in all office visits were a treatment was reported.

Given the high prevalence of hypertension and its burden in the United States, antihypertensive agents will continue to play an increasingly important role in clinical practice. Our data suggest the landscape of hypertension treatment has changed substantially since 1997. Although an increasing number of non-branded products are available, a growing prevalence of hypertension and continued undertreatment of many subjects suggest that the large public health burden from this common disease is unlikely to abate.
